# Knowledge, Attitudes, and Practices Regarding Refractive Eye Surgeries Among Adults in the United Arab Emirates

**DOI:** 10.7759/cureus.94153

**Published:** 2025-10-08

**Authors:** Hashim S Almishhadany, Rita Alkhatib, Yasmeen Saleh, Ahmad Alshouraa, Hussain Almishhadany, Hiba Barqawi, Eman Abu Gharbieh

**Affiliations:** 1 Emergency Medicine, Emirates Health Services, Sharjah, ARE; 2 General Practice, University of Sharjah, Sharjah, ARE; 3 Family Medicine, Dubai Health, Dubai, ARE; 4 Medicine, University of Sharjah, Sharjah, ARE; 5 Medicine, Ajman University, Ajman, ARE; 6 Clinical Sciences, University of Sharjah, Sharjah, ARE

**Keywords:** attitude, knowledge, lasik, practice, public perception, refractive errors, refractive surgery, smile, uae, vision correction

## Abstract

Background and aim

Refractive errors (REs) are a leading cause of visual impairment globally. Surgical correction methods like laser-assisted in situ keratomileusis (LASIK), laser-assisted subepithelial keratectomy (LASEK), and small-incision lenticule extraction (SMILE) are available in the United Arab Emirates (UAE), but public acceptance and understanding remain inconsistent. This study aims to assess knowledge, attitudes, and practices (KAP) regarding refractive surgery among adults in the UAE and to explore demographic and experiential factors influencing surgical uptake and perception.

Methods

A cross-sectional, questionnaire-based study targeting UAE residents aged 18 and above was designed, consisting of a self-administered online survey, available in English and Arabic. It was used to gather data on demographics, vision correction behaviors, awareness of refractive procedures, and surgery-related experiences and beliefs. Descriptive and bivariate analyses were conducted using SPSS v26. Statistical significance was set at p ≤ 0.05.

Results

A total of 495 valid responses were analyzed. Over half of the participants (54.75%) had refractive errors, predominantly myopia. Awareness of refractive surgery was high (80%), with LASIK being the most familiar procedure. However, only 27.27% had undergone surgery. Among them, 70.37% reported full satisfaction, while dry eye and visual miscorrection were the most cited complications. Attitudes varied: 53.33% perceived surgery as a cure, while 35.76% expected reduced dependence on corrective lenses. Perceived safety and recovery expectations significantly predicted willingness to undergo or recommend surgery. Younger age, female gender, and higher education levels were associated with more favorable views. Multivariate analyses revealed that knowledge alone was insufficient to drive uptake. Satisfaction with outcomes, trust in providers, and perceptions of safety were more influential. Information sourced from ophthalmologists or educational institutions was linked to more accurate expectations, while social media and peer influence were associated with uncertainty or misconceptions.

Conclusions

Although awareness of refractive surgery is widespread among UAE adults, misconceptions, safety concerns, and variable trust in the procedure limit its broader acceptance. Tailored public health messaging, professional-led education, and improved access to evidence-based information are critical to addressing these barriers. Understanding the sociocultural and psychological drivers behind surgical decisions can inform strategies to enhance informed uptake and satisfaction with refractive care in the UAE and similar contexts.

## Introduction

Refractive errors (REs), including myopia, hyperopia, astigmatism, and presbyopia, are among the most common causes of visual impairment globally. An estimated 2.2 billion people are affected by vision impairment, with at least one billion of these cases being preventable or uncorrected [1]. While corrective options such as spectacles, contact lenses, and surgical procedures are widely available and effective, disparities in awareness, access, and acceptance of refractive correction remain prominent, particularly in developing countries and urbanizing populations [2].

Several studies from the Middle East and Africa highlight considerable gaps in awareness and attitudes regarding refractive surgery. In Saudi Arabia, cross-sectional surveys found that many adults had limited knowledge of laser procedures, with misconceptions and safety concerns being common barriers to acceptance [3,4]. In Ethiopia, a study among school teachers revealed that although spectacles were familiar, awareness of surgical options was low and associated with socioeconomic disparities [5]. Similarly, in Iraq, a study among Baghdad medical students found low levels of knowledge and substantial uncertainty about the safety and benefits of refractive procedures [6].

Globally, similar trends have been documented. A study in Krachi, Ghana, found that although medical students were generally aware of refractive surgery, confidence in its safety and indications varied [7]. In India, multiple studies across Kanyakumari and Lucknow revealed that while undergraduate students and healthy adults were familiar with refractive error correction, surgical awareness was inconsistent and often influenced by rural versus urban residence [8,9]. An Italian study among optometry students further highlighted significant gaps in procedural understanding despite high theoretical exposure [10]. The United Arab Emirates (UAE), a rapidly developing and highly urbanized nation, is experiencing a growing prevalence of refractive errors, particularly among youth and digital device users. Refractive surgical procedures such as laser-assisted in situ keratomileusis (LASIK), laser-assisted subepithelial keratectomy (LASEK), and small-incision lenticule extraction (SMILE) are available and increasingly popular for permanent vision correction. These methods have well-established safety and efficacy profiles [11]. However, despite the accessibility of advanced ophthalmic care, no comprehensive study to date has evaluated public knowledge, attitudes, and practices (KAP) toward refractive surgery within the UAE. Given the UAE’s multicultural population and high reliance on private healthcare services, public perceptions of elective eye surgery may differ from regional or international trends. Understanding the KAP of refractive surgery in this unique context is essential to guide public health messaging, dispel myths, and enhance informed decision-making. This study aims to fill that gap by evaluating the KAP regarding surgical correction of refractive errors among adults in the UAE and identifying key demographic and experiential factors influencing uptake and perception.

## Materials and methods

This cross-sectional study assessed knowledge, attitudes, and practices (KAP) regarding refractive surgery among adults in the United Arab Emirates (UAE). Data were collected at a single time point using a structured, self-administered online questionnaire. The target population included UAE residents aged 18 and above with internet access and technological literacy. Inclusion criteria were age ≥18, UAE residency, and fluency in English or Arabic. The survey platform automatically excluded those under 18, not residing in the UAE, or submitting incomplete/inconsistent responses.

The sample size was calculated using the Raosoft calculator (Raosoft, Inc, Seattle, Washington, United States) by the standard formula for proportions: n = Z²·p(1-p)/e², with finite population correction n_adj = (N·n)/((N - 1) + n). For a population of 20,000, 95% confidence level (Z = 1.96), 5% margin of error (e = 0.05), and response distribution of 50% (p = 0.5), this yields 377 participants. An additional 10% was added to account for potential exclusions [12]. Non-probability volunteer sampling was used, with recruitment via social media. The data collection instrument, made in both English and Arabic versions, comprised 45 close-ended items across two sections: demographics (age, gender, nationality, education, marital status, occupation) and KAP (awareness of procedures like LASIK/SMILE, perceptions of safety/effectiveness, prior experiences, and willingness to undergo or recommend surgery). It was adapted from validated tools used in similar studies [6,13].

The psychometric properties of the study tool were established through several steps. Content validity was ensured by adapting items from previously published KAP studies on refractive surgery and reviewing them with experts in ophthalmology and public health [6,13]. The questions were further assessed by researchers experienced in KAP methodology to confirm their relevance and clarity. Construct validity was addressed by organizing the questionnaire into the three core domains of knowledge, attitude, and practice, ensuring that each item measured its intended construct. Finally, the tool was pilot tested over two weeks among a representative subset of participants to evaluate comprehension, timing, and consistency, with minor modifications made accordingly. The final survey was distributed through Instagram, WhatsApp, email, and Twitter over a period of six months in 2024-2025. Participants gave digital informed consent, and the average completion time was five to six minutes. Responses were recorded securely online. Ethical approval was obtained from the University of Sharjah Research Ethics Committee, Sharjah, UAE (approval: REC-24-06-03-01-SE, 18-09-2024), and participation was voluntary, anonymous, and confidential.

Data were analyzed using IBM SPSS Statistics for Windows, Version 26.0 (IBM Corp., Armonk, NY, USA). Descriptive statistics were calculated as frequencies and percentages for categorical variables and summarized demographics and KAP trends. Inferential analyses included chi-square tests to assess associations between variables. Odds ratios (ORs) with corresponding 95% confidence intervals (CIs) were computed to estimate the strength of associations. A p-value of <0.05 was considered statistically significant.

## Results

Demographics

After filtering out invalid responses, a total of 495 participants were included. Females constituted 53.33% (n = 264/495) of the sample. The largest age group was 18-25 years at 34.95% (n = 173/495). A summary of the respondents’ demographics is shown in Table 1.

**Table 1:** Demographics of respondents.

Demographics	Demographic value	Number/495	Percentage
Gender	Male	231	47.7%
	Female	264	53.3%
Age	18-25	173	35.0%
	26-39	117	23.6%
	40-59	137	27.6%
	60+	68	13.7%
Highest degree obtained	Middle school or lower	22	4.4%
	High school	93	18.8%
	Diploma/Bachelor's	223	45.1%
	Postgraduate	157	31.7%
Marital status	Married	182	36.8%
	Single	165	33.3%
	Divorced	114	23.0%
	Widowed	34	6.9%
Nationality	UAE National	149	30.1%
	Other Non-UAE Arab	216	43.6%
	Non-Arab	130	26.3%
Occupation	Healthcare worker	144	29.1%
	Non-healthcare worker	110	22.2%
	Health sciences student	93	18.8%
	Non-health sciences student	31	6.3%
	Unemployed (non-health)	45	9.1%
	Unemployed (healthcare)	32	6.5%
	Housewife	40	8.1%

Prevalence of refractive error

**Figure 1:**
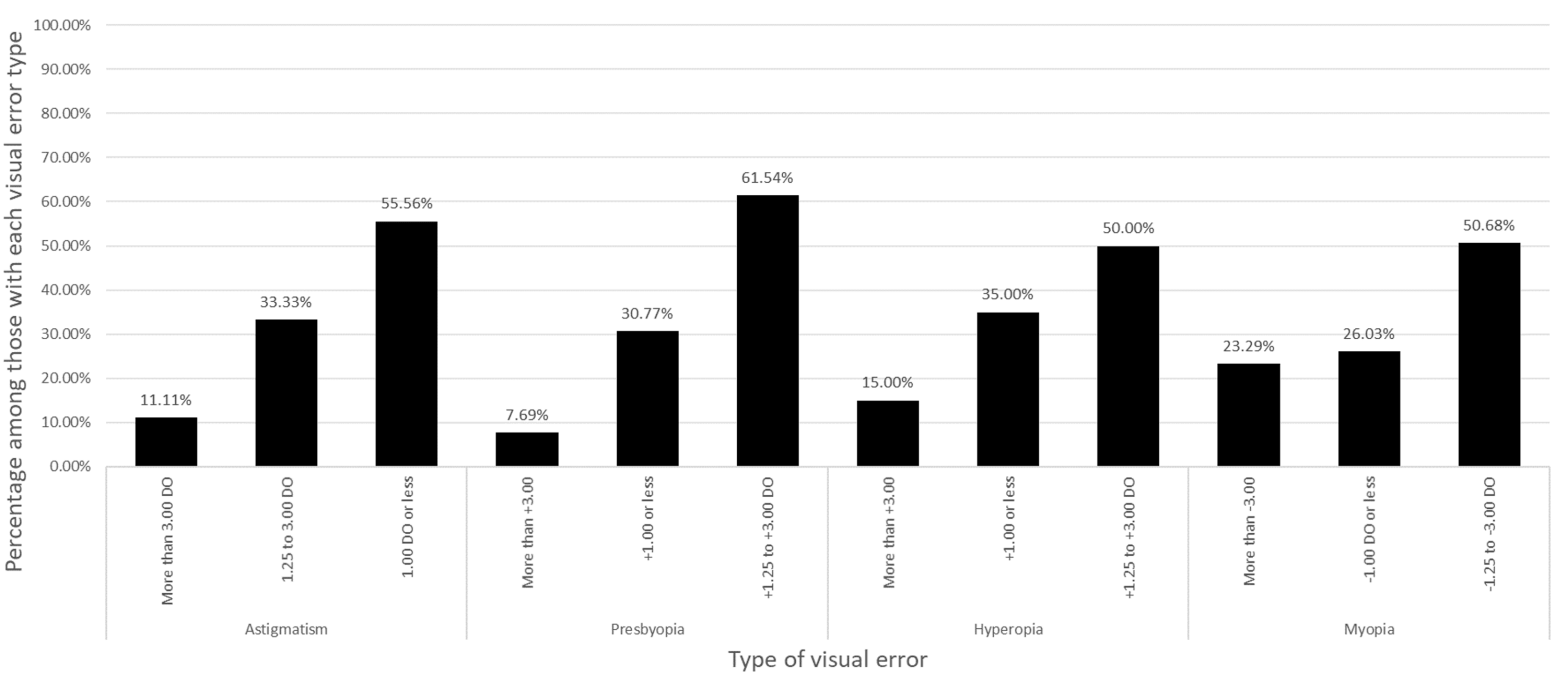
Percentage distribution of refractive error severity levels across different refractive error types among study participants. This figure illustrates the severity levels of different refractive error types, including astigmatism, myopia, hyperopia, and presbyopia and varying degrees of diopters (DO). Percentages were calculated based on the proportion of respondents with each severity level. The chart highlights the distribution of mild, moderate, and severe refractive errors. Data are expressed as percentages of total valid responses.

**Figure 2:**
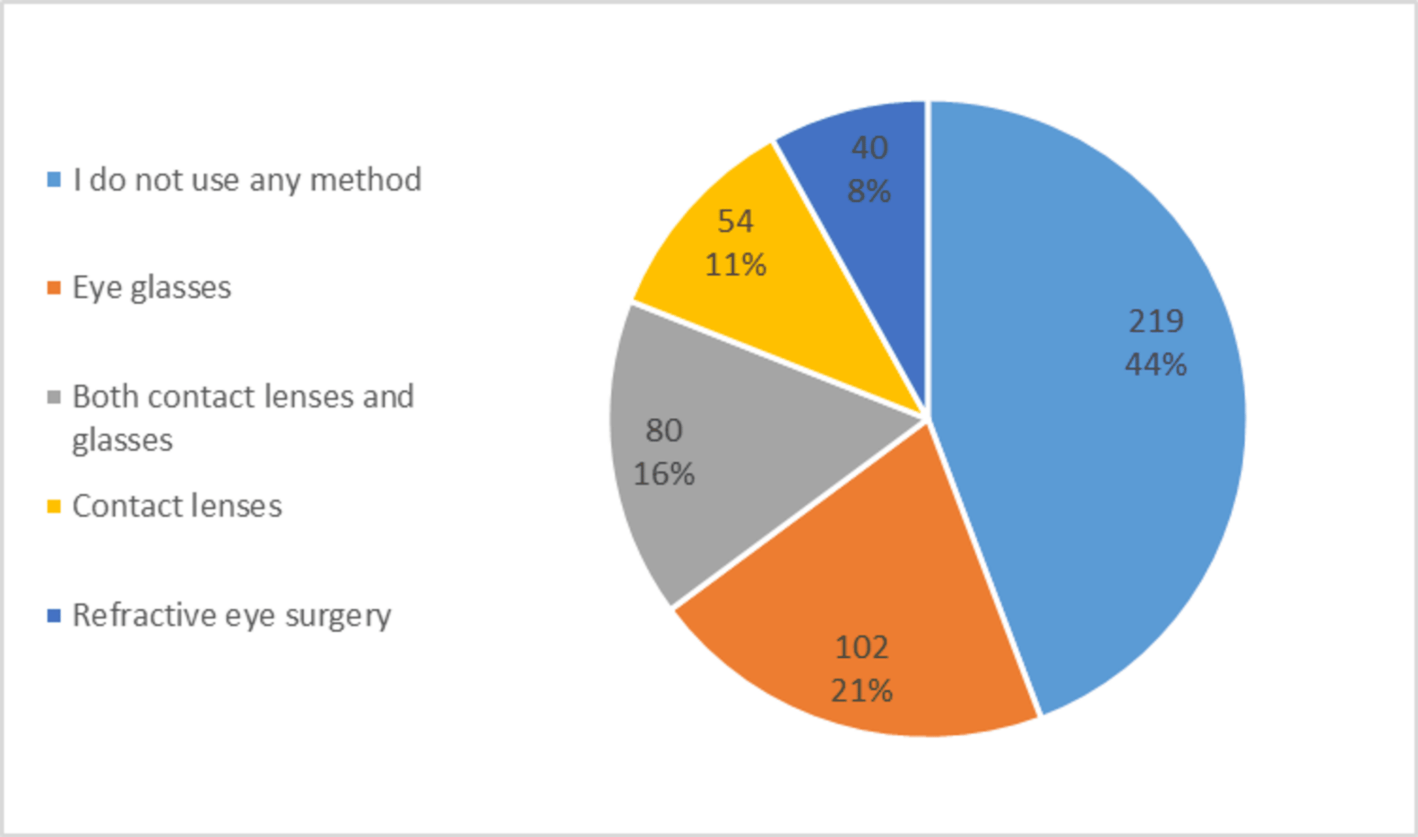
Methods of vision correction reported by study participants in the UAE. This pie chart illustrates the distribution of vision correction methods among participants (n = 495). Each slice displays both the frequency (n) and percentage (%) for eyeglasses, contact lenses, combined glasses and lenses, refractive surgery, and no correction. Eyeglasses were the most common method, while refractive surgery accounted for a smaller proportion. Percentages are based on the total sample size.

As for the refractive error prevalence, more than half of the participants, 54.75% (n = 271/495), reported having a refractive error. Among them, nearsightedness (myopia) was most common at 46.13% (n = 125/271), followed by farsightedness at 23.62% (n = 64/271), astigmatism at 22.87% (n = 62/271), and presbyopia at 15.13% (n = 41/271). Most of these respondents were diagnosed between 18 and 39 years (31.0%, n = 84/271). As for the severity of the refractive errors, Figure 1 shows the percentage of each severity level by refractive error type. As for vision correction, its usage was reported by 59.39% (n = 294/495). The distribution of the methods used is shown in Figure 2.

Awareness of refractive surgery

Awareness of refractive surgery was high, with 80.0% (n = 396/495) indicating awareness. The most common sources were family and friends (50.1%, n = 248/495), university or school (37.78%, n = 187/495), ophthalmologists (35.96%, n = 178/495), and social media (34.14%, n = 169/495), while other doctors (non-ophthalmologists) only had 23% (n = 114/495) as a source of awareness. The most familiar procedure was LASIK (60.81%, n = 301/495), followed by SMILE (30.51%, n = 151/495), LASEK (29.9%, n = 148/495), photorefractive keratectomy (PRK) (27.68%, n = 137/495), and Femto-LASIK (24.04%, n = 119/495). When asked about the safest procedure, 33.74% (n = 167/495) responded “I don’t know,” followed by LASIK (20.4%, n = 101/495), SMILE (13.74%, n = 68/495), Femto-LASIK (11.31%, n = 56/495), PRK (10.71%, n = 53/495), and LASEK (10.1%, n = 50/495). Regarding complications, 26.06% (n = 129/495) were unaware of any, while others reported dry eyes at 34.95% (n = 173/495), undercorrection at 34.34% (n = 170/495), halos or double vision at 32.32% (n = 160/495), overcorrection at 29.29% (n = 145/495), astigmatism at 28.08% (n = 139/495), flap problems at 25.66% (n = 127/495), and corneal ectasia at 15.56% (n = 77/495).

Attitudes and beliefs regarding refractive surgery

Regarding expectations, 53.33% (n = 264/495) viewed refractive surgery as a definitive cure, while 35.76% (n = 177/495) expected reduced dependence on glasses or lenses. Overall, 27.27% (n = 135/495) had undergone refractive surgery. Their main reasons included myopia at 35.56% (n = 48/135), hyperopia at 17.78% (n = 24/135), presbyopia at 14.81% (n = 20/135), and astigmatism at 14.07% (n = 19/135). Satisfaction was high: 70.37% (n = 95/135) were fully satisfied, 24.44% (n = 33/135) partially satisfied, and 5.19% (n = 7/135) unsatisfied. Most surgeries occurred at ages 18-39 (29.63%, n = 40/135). Among those who had surgery, 64.44% (n = 87/135) would recommend it. Reported complications included dry eye at 25.93% (n = 35/135), keratitis at 17.04% (n = 23/135), inappropriate correction at 13.33% (n = 18/135), and no complications in 56.3% (n = 76/135). Among all respondents, 70.51% (n = 349/495) believed dry eye can occur post-surgery; 54.34% (n = 269/495) considered it temporary, and 27.07% (n = 134/495) permanent. LASIK was seen as the most likely to cause dry eye (18.99%, n = 94/495). Additionally, 63.03% (n = 312/495) believed surgery can be performed on children, and 38.38% (n = 190/495) thought multiple sessions might be needed. In terms of perceived outcomes, 24.85% (n = 123/495) rated surgery very effective and 52.93% (n = 262/495) effective. For safety, 22.02% (n = 109/495) said very safe, 46.26% (n = 229/495) safe, 25.66% (n = 127/495) somewhat safe, and 6.06% (n = 30/495) not safe. Additionally, 64.44% (n = 319/495) believed recovery is short, and 77.78% (n = 385/495) thought vision might weaken again after surgery.

Practices regarding refractive surgery

Interest in undergoing surgery in the future was reported by 62.42% (n = 309), and 66.87% (n = 331) expressed a desire to learn more about refractive procedures. Barriers among those willing but yet to undergo surgery included good current vision (15.86%, n = 56), low visual deficit (9.92%, n = 35), medical contraindications (6.8%, n = 24), fear of complications (12.75%, n = 45), and financial constraints (11.61%, n = 41). Others cited lack of opportunity (12.18%, n = 43), confidence in the procedure (9.92%, n = 35), or in doctors (5.38%, n = 19) as deterrents. Among those unwilling to undergo surgery, the top reasons included fear of complications (16.71%, n = 59/353), low perceived need (12.18%, n = 43/353), lack of knowledge (13.31%, n = 47/353), and financial cost (15.86%, n = 56/353). A small portion also cited lack of confidence in the procedure (7.93%, n = 28/353) or medical advice against it (7.08%, n = 25/353).

Multivariate analysis

As for the multivariate analysis, gender was found to be associated with the choice of corrective method use (χ² = 10.26, p = 0.0363) and the belief that corrective eye surgery can be performed on children (χ² = 5.8, p = 0.0161). Overall, males were more likely than females to use no vision correction. A higher proportion of males (68.83%) believed that corrective eye surgery can be performed on children. Awareness of surgery for correcting refractive errors varied by age group (χ² = 30.6, p < 0.001). The highest awareness was among the 18-25 years age group, being 5.70 times more likely to be aware than the 26-39 years age group. Age was also significantly linked to willingness to undergo surgery (χ² = 32.1, p < 0.001). The 26-39 group had the highest combined willingness or experience at 82.05% having either undergone or willing to undergo refractive surgery. Perceived safety also varied by age (χ² = 20.9, p = 0.0131), with only 2.31% of those aged 18-25 viewing it as unsafe. Education level significantly affected unwillingness to undergo surgery (χ² = 23.0, p < 0.001); those with middle school education or lower were the most unwilling. Nationality was also significant (χ² = 17.6, p = 0.0015), with UAE nationals showing the highest unwillingness.

Beliefs about safety and outcomes were strong predictors of willingness to undergo refractive surgery. Believing the procedure was unsafe was significantly linked to reluctance (χ² = 12.6, p = 0.0498). Among those who believed it was safe, 45.28% were willing to undergo it and were 2.07 times more likely to do so than those who believed it was unsafe. Recovery period perceptions also showed a significant association (χ² = 9.92, p = 0.007). Willingness was 39.81% among those who believed recovery was short, compared to 35.8% among those who thought it was long. Perceptions of outcomes and potential relapse were similarly significant (χ² = 12.7, p = 0.0128). A strong link existed between refractive error presence and willingness to undergo surgery if needed (χ² = 117.38, p < 0.001). Unwillingness was 23.99% among those with refractive errors versus 43.75% among those without. Frequency of correction method use also had a significant impact (χ² = 11.63, p = 0.003).

**Figure 3:**
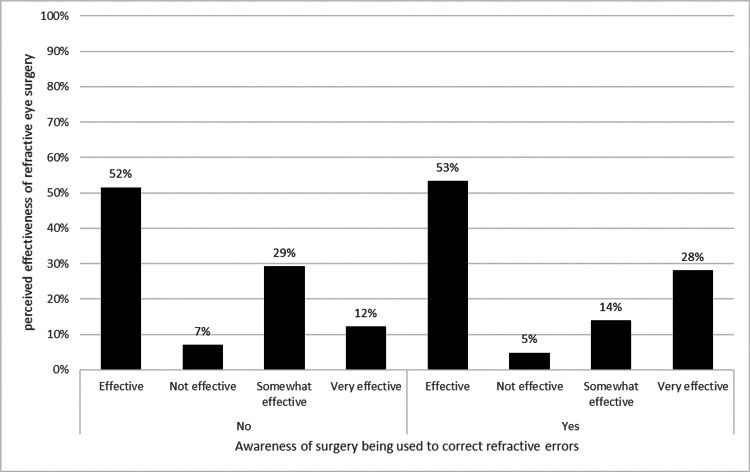
Association between refractive surgery awareness and perceived effectiveness for vision correction. This figure shows participants’ awareness of refractive eye surgery and how they perceive its effectiveness, categorized as “not effective,” “somewhat effective,” “effective,” or “very effective.” Percentages are based on the total number of responses within each awareness group (n = 495). Chi-square testing was used to assess the significance of the association (p < 0.05 considered significant).

**Figure 4:**
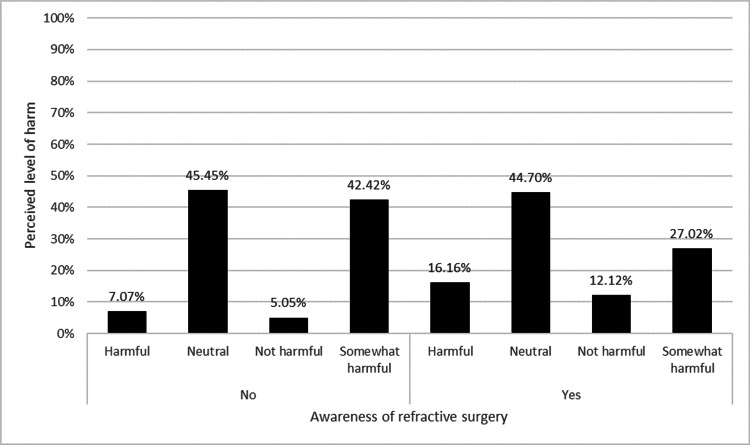
Association between refractive surgery awareness and perceived level of harm associated with the procedure. This figure demonstrates how awareness of refractive surgery relates to perceived harm, with categories ranging from “not harmful” to “harmful.” Percentages reflect the distribution within each awareness category. Statistical analysis was performed using chi-square tests to determine whether perceptions of harm were significantly associated with awareness levels (p < 0.05 considered significant).

Awareness of refractive surgery significantly influenced belief in its effectiveness (χ² = 19.97, p < 0.001), as shown in Figure 3. Those unaware were 1.51 times more likely to believe it was ineffective. Among those aware, 73.48% acknowledged dry eye as a potential postoperative issue, compared to 58.59% of those unaware (χ² = 7.75, p = 0.0054). Awareness also significantly affected perceptions of potential harm (χ² = 14.5, p = 0.0023), with distributions shown in Figure 4. Additionally, recognizing dry eye as a postoperative issue was significantly associated with perceived safety (χ² = 8.7, p = 0.0337); 71.06% of those acknowledging dry eye still considered the procedure safe or very safe.

Satisfaction with surgical outcomes significantly predicted the likelihood of recommending refractive surgery (χ² = 21.4, p < 0.001). Willingness to recommend was 71.58% among satisfied participants, 51.52% among those partially satisfied, and 28.57% among those dissatisfied. Postoperative dry eye was also a significant predictor (χ² = 37.6, p < 0.001). Among those without dry eye, 78.00% would recommend surgery and were 10.25 times more likely to do so than those who experienced it. Consistent use of vision correction significantly predicted undergoing or willingness to undergo surgery (χ² = 11.6, p = 0.003); 73.98% of consistent users had done or would consider the procedure. Perceived inappropriate correction strongly influenced recommendations (χ² = 16.4, p < 0.001). Among those without this issue, 70.94% would recommend surgery, compared to 22.22% of those who experienced it (OR = 8.54). Postoperative infection also had a significant impact (χ² = 10.8, p = 0.0046). Willingness to recommend was 67.19% among those without infection, but only 14.29% among those who developed one (OR = 12.29).

## Discussion

This study represents the first comprehensive KAP assessment of refractive surgery in the UAE, offering baseline data for policy and future research. The large sample size and detailed evaluation reveal that while awareness is high, understanding and decision-making remain inconsistent, mirroring global trends in health literacy and elective procedures [14]. The UAE’s technologically advanced, multicultural healthcare system likely contributes to the high overall awareness.

Awareness significantly influenced participants’ beliefs. Those familiar with refractive procedures viewed them as effective and recognized complications like dry eye, in line with previous studies [6,7]. The source of information was critical: individuals advised by ophthalmologists or academic bodies had greater confidence and more accurate expectations than those informed by peers or media, emphasizing the importance of professional guidance to reduce misconceptions [5,8]. Nonetheless, superficial knowledge persisted, especially uncertainty over which procedure is safest, echoing trends from other Middle Eastern contexts [3]. This underscores the need for targeted education on safety and efficacy.

Age influenced attitudes in nuanced ways. Young adults showed high awareness but lower willingness to undergo surgery, possibly due to conflicting online narratives fostering hesitancy. Older adults seemed more motivated by functional needs than information. Education also significantly affected decision-making; higher educational attainment correlated with greater willingness, while lower education linked to hesitancy, likely reflecting limited health literacy and perceived barriers like access and cost. This supports the Theory of Planned Behavior, which emphasizes perceived control and autonomy in health decisions [15]. Public misconceptions were widespread. Over half believed dry eye post-surgery to be temporary, although some correctly acknowledged its potential permanence, aligning with clinical realities [16]. Many also mistakenly thought refractive surgery was suitable for children or required multiple primary sessions, revealing a gap between surface awareness and detailed understanding.

Patient satisfaction strongly influenced willingness to recommend surgery, confirming that personal outcomes shape public attitudes [7]. Complications, especially dry eye and visual miscorrection, reduced advocacy, highlighting the need for effective postoperative care [16]. Interestingly, awareness of dry eye risk alone did not reduce perceived safety, suggesting that expectation management may ease concerns. Trust in healthcare providers was pivotal, consistent with broader trends in elective care engagement [17]. This emphasizes that awareness alone may not suffice; satisfaction and trust remain critical factors for uptake.

Findings closely align with other KAP studies regionally and globally. In Saudi Arabia, similar patterns of high awareness and persistent misconceptions were observed [3,4]. In our study, nearly half of those aware of refractive surgery had no intention to undergo it, suggesting that psychological, cultural, and socioeconomic factors, also noted in Middle Eastern and sub-Saharan African populations, are at play [6,18].

The UAE context, however, revealed unique hesitancy among nationals, diverging from regional patterns and suggesting deeper cultural or trust-related influences. Unlike other regions where cost is a key barrier, UAE participants more often cited fear of complications or satisfaction with current vision [18]. This may reflect the country’s higher socioeconomic status and advanced healthcare access, which reshape decision-making around elective care. Hence, tailored strategies should address emotional, cultural, and trust-based barriers unique to the UAE.

Understanding KAP dynamics is globally important, as refractive errors remain a major cause of visual impairment, worsened by misinformation and affordability concerns [19]. Refractive surgery, often elective and private-sector-driven, is seen as cosmetic rather than essential. Public health initiatives should reframe it as a crucial vision-health intervention that enhances productivity and life quality [11].

Our findings show that patients without complications often report high satisfaction and recommend surgery. However, advocacy dropped sharply among those who experienced complications, underscoring the need for careful patient selection, surgical precision, and strong postoperative care to maintain public confidence. Improving uptake requires enhancing the quality and reach of professional medical information, addressing specific concerns about complications, and developing culturally sensitive educational materials for the UAE’s diverse population. Regular users of corrective eyewear are particularly promising targets, given their awareness of the limitations of glasses or contacts.

This study has limitations. First, the use of online non-probability sampling introduced potential selection bias; respondents were typically younger, better educated, and more digitally engaged. Second, self-reported data may be influenced by recall or social desirability bias, particularly concerning satisfaction and complications. Third, the cross-sectional design limits causal inference. Future regression analyses are needed to control for confounding variables [20].

Future studies should adopt representative sampling, including older adults and lower-income groups, to improve generalizability [21]. Longitudinal designs could help determine whether intentions lead to action [22]. Qualitative approaches like focus groups could provide deeper insights into emotional and cultural drivers of elective decisions. Co-designed interventions with healthcare professionals should also test educational strategies and tools to enhance decision-making and combat misinformation [23]. Moreover, cost-effectiveness analyses comparing surgery with long-term eyewear use could inform equitable access policies [19]. Finally, future intervention studies should test various educational formats, information sources, and decision-making tools to improve understanding of refractive surgery, offering evidence-based guidance for both clinicians and public health policymakers.

## Conclusions

This study highlights the complexity of public engagement with refractive eye surgery in the UAE. While awareness is generally high, knowledge gaps, safety concerns, and trust issues remain significant obstacles to uptake. Factors such as age, gender, education, nationality, and prior satisfaction with surgery play critical roles in shaping individual decisions. Importantly, this research underscores that awareness alone is not a reliable predictor of acceptance or practice. Instead, patient confidence, rooted in perceived safety, trusted healthcare interactions, and satisfactory outcomes, is more influential. Health communication efforts should therefore go beyond information dissemination to actively build trust, dispel myths, and address practical barriers such as cost and fear of complications. To ensure equitable and informed access to refractive surgical care, public health strategies must be culturally tailored, data-driven, and inclusive of diverse voices. As refractive error prevalence continues to rise in the digital age, empowering individuals with accurate, contextualized knowledge and accessible surgical options will be essential to reducing avoidable visual impairment in the UAE and beyond.
